# Reactive oxygen production induced by near-infrared radiation in three strains of the Chl *d*-containing cyanobacterium *Acaryochloris* *marina*

**DOI:** 10.12688/f1000research.2-44.v2

**Published:** 2013-03-13

**Authors:** Lars Behrendt, Marc Staal, Simona M Cristescu, Frans JM Harren, Martin Schliep, Anthony WD Larkum, Michael Kühl

**Affiliations:** 1 Marine Biology Section, Department of Biology, University of Copenhagen, Helsingør, DK-3000, Denmark; 2 Section for Microbiology, Department of Biology, University of Copenhagen, Sølvgade, DK-1307, Denmark; 3 Life Science Trace Gas Facility, Radboud University Nijmegen, Heyendaalseweg, 6525 AJ, Netherlands; 4 Plant Functional Biology and Climate Change Cluster, School of the Environment, University of Technology Sydney, Sydney, NSW 2007, Australia; 5 Singapore Centre on Environmental Life Sciences Engineering, School of Biological Sciences, Nanyang Technological University, Nanyang, Singapore

## Abstract

Cyanobacteria in the genus *Acaryochloris *have largely exchanged Chl *a* with Chl *d,* enabling them to harvest near-infrared-radiation (NIR) for oxygenic photosynthesis, a biochemical pathway prone to generate reactive oxygen species (ROS). In this study, ROS production under different light conditions was quantified in three *Acaryochloris* strains (MBIC11017, HICR111A and the novel strain CRS) using a real-time ethylene detector in conjunction with addition of 2-keto-4-thiomethylbutyric acid, a substrate that is converted to ethylene when reacting with certain types of ROS. In all strains, NIR was found to generate less ROS than visible light (VIS). More ROS was generated if strains MBIC11017 and HICR111A were adapted to NIR and then exposed to VIS, while strain CRS demonstrated the opposite behavior. This is the very first study of ROS generation and suggests that *Acaryochloris* can avoid a considerable amount of light-induced stress by using NIR instead of VIS for its photosynthesis, adding further evolutionary arguments to their widespread appearance.

## Introduction

Most oxyphototrophs use visible light (VIS, 400–700 nm) for chlorophyll (Chl) *a*-driven photosynthesis, while cyanobacteria in the genus *Acaryochloris* largely employ Chl *d*, thereby enabling them to use near-infrared radiation (NIR, >700 nm) for oxygenic photosynthesis ^1, 2^. Two of the strains are well described in their growth and photopigment composition: The type strain *Acaryochloris marina* MBIC11017 was isolated from the didemnid ascidian *Lissoclinum patella* from coral reefs habitats in Palau ^2, 3^ and was later genome sequenced ^4^. The other strain, HICR111A, originates from swipes of coral substrate collected on Heron Island, Australia and was also genome sequenced ^5^. Since its first discovery, other *Acaryochloris* strains have been obtained from Japanese macroalgae (strain Awaji ^6^), from surfaces in a hypertrophic lake in the US (strain CCMEE5410 ^7^), and most recently from Australian mangroves ^8^ (strain MPGRS1) and stromatolites in Shark Bay, Western Australia ^9^ (ssball1).

The light microenvironment in natural habitats occupied by *Acaryochloris* spp. has a high contribution of NIR relative to visible light ^10– 12^ and such habitats appear to create similar niche differentiation with bacteria carrying specialized photopigments such as Chl *d*/ *f* or bacteriochlorophylls ^10, 13^. The notion of a global distribution of Chl *d* and cyanobacteria in the genus *Acaryochloris* ^11, 14^ further reinforces the need to obtain information on the photobiology of Chl *d*-containing oxyphototrophs. Understanding the adaptive mechanisms in oxyphototrophs capable of using wavelengths beyond VIS is of interest as it provides information concerning the usability, stress levels and limitations associated with NIR-driven oxygenic photosynthesis.

Of all biological pathways, photosynthetic electron transport is particularly prone to produce reactive oxygen species (ROS) due to the very high (positive) redox potential of the primary donor of photosystem II, needed to oxidize water, and the low redox potential of the primary electron acceptor of photosystem I, needed to reduce ferredoxin; here, singlet oxygen ( ^1^O _2_), is produced by PSII and superoxide anions ( ^-^O _2_)/hydrogen peroxide (H _2_O _2_) in the Mehler, ascorbate peroxidase (MAP) pathway of PSI ^15– 17^. ROS encompasses the production of singlet oxygen, superoxide anions, hydrogen peroxide and hydroxyl radicals (OH), all of which are derived through the local energization of O _2_. If not properly quenched by protective mechanisms, ROS can damage proteins, DNA and other cellular macromolecules, and this damage can ultimately lead to cell death. Known quenching mechanisms encompass enzymes such as superoxide dismutase and catalase or non-enzymatic antioxidants like glutathione, carotenoids and α-tocopherol (vitamin E) ^18^. In plants, ROS and in particular ^1^O _2_ production has been shown to occur at photosystem II upon illumination with visible light ^19, 20^. In cyanobacteria, shorter wavelengths such as ultraviolet radiation (UVR, <400 nm), are known to induce ROS, causing DNA damage, lipid-peroxidation and overall decreased photosynthetic efficiency ^21, 22^. To our knowledge no study has investigated the effect of NIR on ROS production in cyanobacteria.

Relative levels of ROS can be estimated through measurements of e.g. gene expression ^23^, ROS sensitive fluorescence probes ^24^ and enzyme activity ^25^. These methods provide integrated values of ROS production over incubation time intervals ranging from minutes to hours. In this study we used a fast and sensitive laser photo-acoustic gas detector ^26^ that can measure the ethylene produced from the reaction of certain types of ROS with the substrate 2-keto-4-thiomethylbutyric acid (KMBA), previously added to the samples. Such near real-time ROS detection is valuable in determining the immediate effect of treatments on the physiological state and stress level within living organisms. KMBA is thought to diffuse into intact cells ^27^ and, when supplied at saturating concentrations outcompetes other radical scavenging mechanisms. In the KMBA assay, the butyric acid moiety is known to react with the ROS peroxynitrite, hydroxyl radicals and peroxyl radicals ^28^, resulting in the formation of ethylene, which can then be quantified. Whether singlet oxygen and superoxide anions react with KMBA and subsequently form ethylene has not been investigated yet. In other studies, KMBA has been used to test the antioxidant capacity of radical scavengers via their ability to inhibit ethylene formation relative to a control reaction (total oxyradical scavenging capacity, TOSC) ^28, 29^.

In this study, we report the effect of light intensity and spectral composition on ROS generation, as measured in real-time using a laser-photoacoustic gas detector in three different strains of NIR utilizing cyanobacteria belonging to the genus *Acaryochloris*, including a new strain (named *Acaryochloris* CRS), isolated from phototrophic biofilms growing on dead coral branches collected on Heron Island, Australia.

## Results and discussion

**Figure 1.**
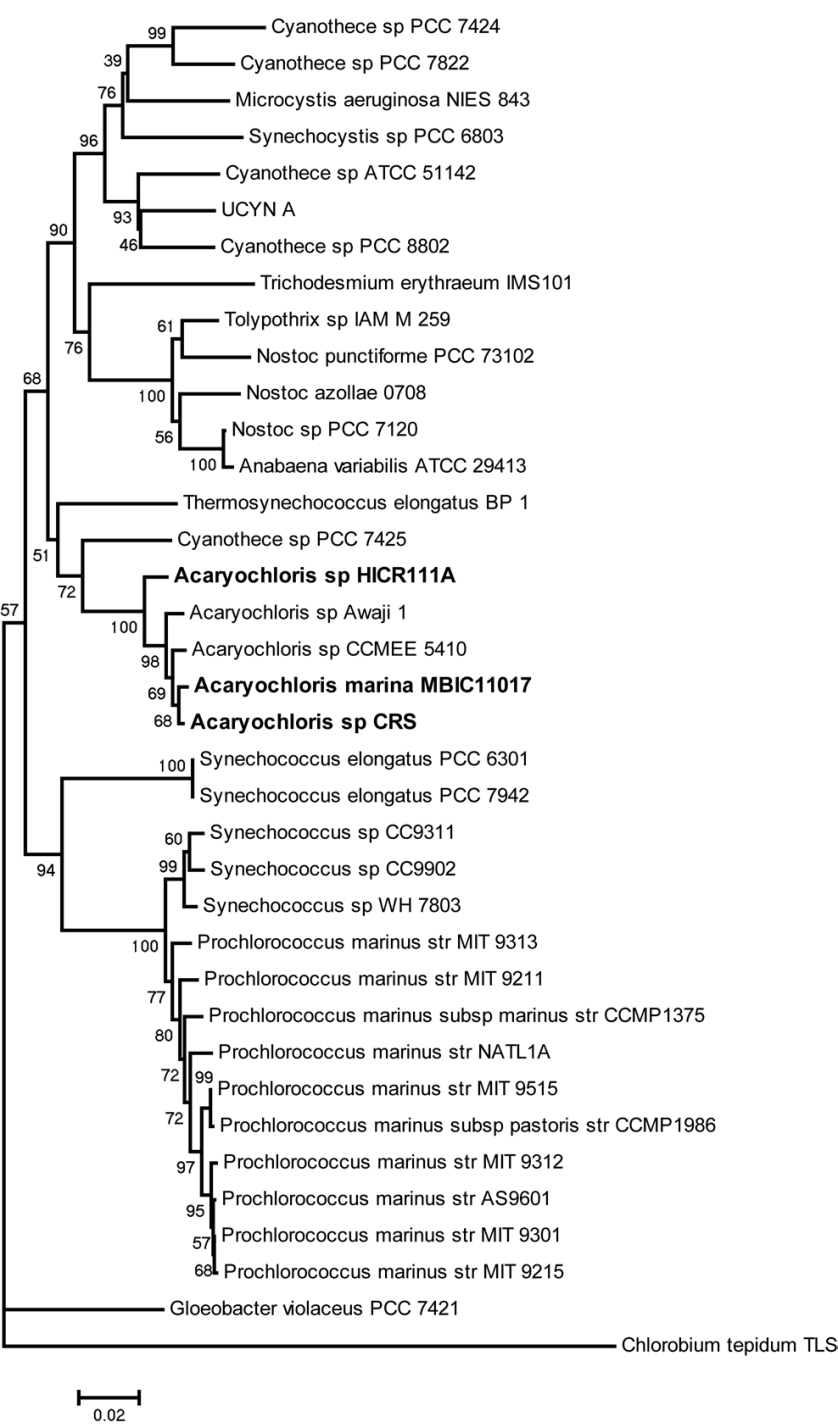
Phylogenetic tree indicating the position of the novel *Acaryochloris* strain CRS based on full-length *16S rRNA* gene sequences. Sequences from other cyanobacteria (35 in total) were obtained from the SILVA database while CRS-specific sequences were obtained through PCR amplification and subsequent sequencing. Phylogeny was calculated using Neighbor-joining methods and Jukes-Cantor substitution models as implemented in MEGA5. Tree stability was assessed using bootstrapping at 10000 replications. Only bootstrap values >50% are displayed within the tree. The scale represents 0.02 substitutions per nucleotide position. The green-sulphur bacterium *Chlorobium tepidum* TLS was chosen as an outgroup.

**Figure 2.**
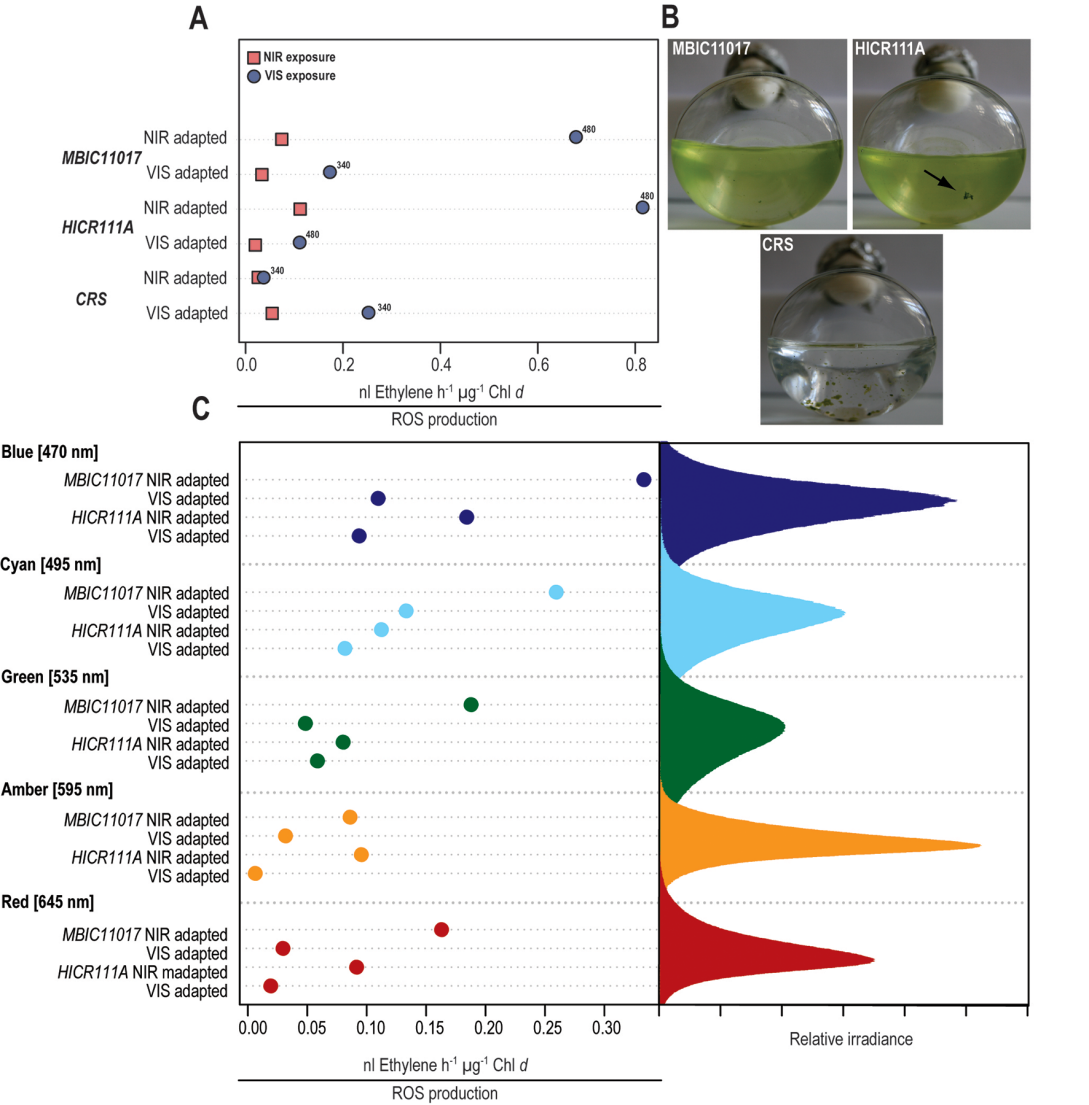
ROS induced ethylene production in the three *Acaryochloris* sp.strains MBIC11017, HICR111A and the novel strain CRS. All values were normalized to Chl *d* concentration as determined by spectrophotometry. All cultures were grown under either near-infrared radiation (NIR, 720 nm) or visible light (VIS, 400–700 nm) before subsequent light exposure. ( **A**) Cleveland dot-plot of ROS levels measured during exposure of VIS or NIR adapted *Acaryochloris* cells to either VIS (blue dots, 340–480 µmol photons m ^-2^ s ^-1^, as denoted on the graph) or NIR (red squares, 400 µmol photons m ^-2^ s ^-1^). ( **B**) Action spectrum of ROS-induced ethylene production in VIS or NIR adapted strains MBIC11017 and HICR111A. Due to lack of sufficient culture material, action spectra were not determined for *Acaryochloris* strain CRS. Peak emissions of the monochromatic LEDs used for illumination were: red (645 nm), amber (595 nm), green (535 nm), cyan (495 nm) and blue (470 nm). The irradiance in this experiment was adjusted to 300 µmol photons m ^-2^ s ^-1^. ( **C**) Growth forms of the different *Acaryochloris* strains MBIC11017, HICR111A and CRS. All strains displayed were grown under NIR and are approximately one week old. Please note the natural formation of biofilms in *Acaryochloris* strain HICR111A (arrow) and CRS.

We aimed to determine the stress levels associated with Chl *d*-driven oxygenic photosynthesis and tested NIR, VIS and more narrow wavebands for their capacity to induce ROS in three strains of *Acaryochloris*: i) The *Acaryochloris marina* type strain MBIC11017 ^2, 3^, ii) strain HICR111A ^5^ and, iii) a novel strain, named CRS, isolated from dead coral branches which, based on *16S rRNA* gene sequencing, grouped within the genus *Acaryochloris* ( Figure 1). *Acaryochloris* strains MBIC11017 and HICR111A are both well described in terms of their photopigmentation, genomic content, ultrastructure and their capability to perform photoacclimation ^4, 5, 30^. To test whether photoacclimation, i.e., light-dependent change in pigment levels, was associated with increased resistance or sensitivity towards ROS, we acclimated the strains to NIR or VIS prior to experiments. By taking advantage of the real-time ROS detection method, we could for the first time demonstrate the immediate effects of NIR, VIS and other wavelengths on the generation of certain ROS within living cyanobacteria. Specifically, we found that:

(i) Depending on strain and previous adaptation, ROS levels were lower in cells exposed to NIR than in those exposed to VIS ( Figure 2A);

(ii) Exposure to shorter wavelengths such as blue and cyan, generated the most ROS in strain MBIC11017 and HICR111A, while less ROS was produced upon exposure to longer wavelengths (green, amber and red) ( Figure 2C).

(iii) Strain CRS generated less ROS upon exposure to VIS when previously acclimated to NIR, while strain MBIC11017 and HICR111A appear more sensitive to VIS when adapted to NIR ( Figure 2A).

ROS-induced ethylene production in the three Acaryochloris sp. strains MBIC11017, HICR111A and the novel strain CRS. (A) Cleveland dot-plot of ROS production (in nl Ethylene h-1 µg-1 Chl d) measured during exposure of VIS or NIR adapted Acaryochloris cells to either VIS (blue dots, 340-480 µmol photons m-2 s-1, as denoted on the graph) or NIR (red squares, 400 µmol photons m-2 s-1). (C) Action spectrum of ROS-induced ethylene production (nl Ethylene h-1 µg-1 Chl d) in VIS or NIR adapted strains MBIC11017 and HICR111A. Due to lack of sufficient culture material, action spectra were not determined for Acaryochloris strain CRS. Peak emissions of the monochromatic LEDs used for illumination were: red (645nm), amber (595nm), green (535nm), cyan (495nm) and blue (470nm). The irradiance in this experiment was adjusted to 300 µmol photons m-2 s-1.

In *Acaryochloris*, VIS irradiance is primarily absorbed by the photopigments Chl *d* (with maximum absorption occurring at 440–470/710 nm), Chl *a* (440–470/675 nm), carotenoids (440–520 nm) and if present, phycobiliproteins (560–650 nm). NIR provides a more targeted stimulation of photosynthesis and is almost exclusively absorbed by Chl *d*. At comparable photon irradiances (VIS= 340–480 µmol m ^-2^ s ^-1^ versus NIR= 400 µmol m ^-2^ s ^-1^), we found that, depending on strain and previous adaptation, ROS levels were lower in cells exposed to NIR than in those exposed to VIS ( Figure 2A). Based on pulse-amplitude modulated (PAM) fluorometry measurements the light intensities used in our experiments are known to saturate relative-electron transport rates in the type strain MBIC11017 ^12^. Photoinhibition is not observed even at higher, photon irradiance, but we hypothesize that prolonged exposure to relatively high-irradiances (10–20 fold more irradiance than during culturing for 15–20 min) could result in the over-reduction of the primary acceptors on the PSI and PSII side resulting in the production of ROS ^15, 23^. This over-reduction could lead to the transfer of electrons to O _2_, the subsequent generation of superoxide radical, followed by their conversion to hydrogen peroxide as well as hydroxyl radicals ^23, 31^. Alternatively, ROS generation could occur via photosensitized light-harvesting pigments ^32^; however, in intact light-harvesting complexes the efficiency of electron transfer towards the reaction centers is usually outcompeting the formation of long-lived (ROS-forming) chlorophyll triplet states ^33^. This appears particularly true for the unique phycobiliprotein antenna rods in *A. marina* MBIC11017, in which excitation electron transfers to PSII were found to be significantly faster than in Chl *a*-containing cyanobacteria ^34^. Additionally, it is known that within light-harvesting complexes carotenoids are outcompeting O _2_ in the de-excitation of triplet chlorophyll states ^35^. All three strains used in the current study were found to contain zeaxanthin ( Table 1) as well as α-carotene, these carotenoids will bring about rapid quenching of excited Chl states and if necessary can also quench singlet oxygen and aid in general non-photochemical quenching ^33, 36^.

**Table 1.** Photopigment content of the three *Acaryochloris* strains MBIC11017, HICR111A and CRS. The strains were adapted to either visible light (VIS) or far-red light (NIR). Photopigments were identified manually from HPLC chromatograms and ratios calculated based on the derived peak areas. Average values and standard error from the mean from two independent growth experiments are displayed.

	*Acaryochloris* strain					
	MBIC11017		HICR111A		CRS	
**Pigment ratio**	*NIR adapted*	VIS adapted	NIR adapted	VIS adapted	NIR adapted	VIS adapted
**Chl a/d**	0.035 ± *0.002*	0.032 ± *0.005*	0.047 ± *0.007*	0.034 ± *0.001*	0.055 ± *0.022*	0.069 ± *0.039*
**Chl d/zeaxanthin**	2.229 ± *0.845*	3.169 ± *0.224*	2.422 ± *0.691*	3.438 ± *0.037*	2.209 ± *0.123*	NA
**Chl d/α-carotene**	1.694 ± *0.304*	2.248 ± *0.394*	1.907 ± *0.384*	2.262 ± *0.065*	2.484 ± *0.387*	2.750 ± *0.333*
**Zeaxanthin/α-carotene**	0.827 ± *0.177*	0.704 ± *0.075*	0.808 ± *0.072*	0.658 ± *0.026*	1.138 ± *0.238*	0.507 ± *0.341*

Exposure to shorter wavelengths, such as blue (470 nm) and cyan (495 nm) light generated the most ROS in strain MBIC11017 and HICR111A, while less ROS was produced upon exposure to longer wavelengths (green, amber and red) ( Figure 2C). Blue and cyan light-induced ROS production in MBIC11017 and HICR111A is probably due to spectral overlap with the Soret-band absorption of Chl *a*/ *d* (440–470 nm) ( Figure 2B) and the above-mentioned mechanisms in ROS generation. Red (645 nm), amber (595 nm) and green (535 nm) light overlaps with the absorption spectra of phycobiliproteins which, if present, aid in light harvesting and excitation energy transfer towards the photosystems ^34^. Strain MBIC11017 is known to express the phycobiliproteins phycocyanin and allophycocyanin ^37^, while strain HICR111A reportedly lacks phycobiliproteins ^5^.

**Figure 3.**
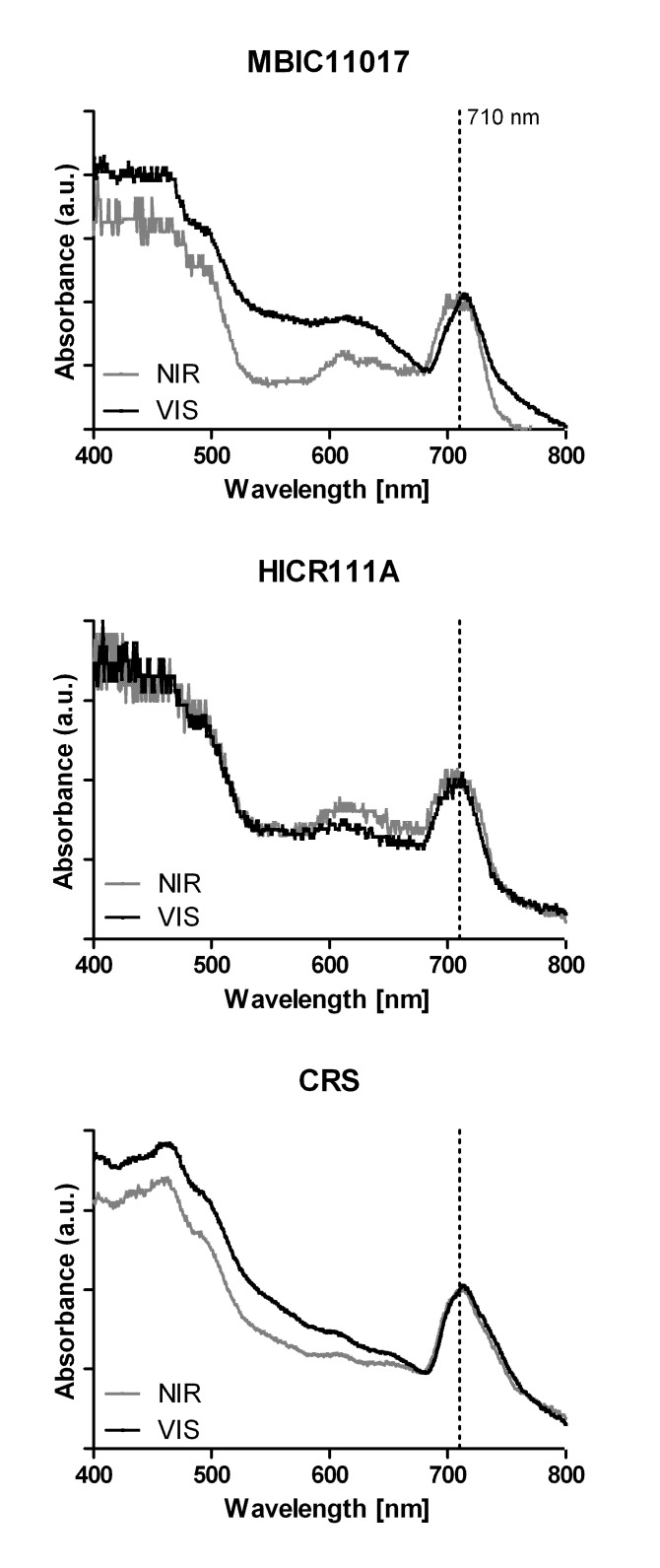
*In vivo* absorption spectra of the three *Acaryochloris* strains MBIC11017, HICR111A and CRS. All strains were adapted to either visible light (VIS) or near infrared radiation (NIR) prior to measurements. All spectra were normalized to the maximal absorbance of Chl *d* at 710 nm.

Comparable ROS levels were observed in strain HICR111A and MBIC11017 under yellow and green light, suggesting the presence of pigments absorbing these wavelengths or the possibility of other light-induced ROS production mechanisms. Spectrophotometric analysis of the strains showed weak absorption in the phycobiliprotein-specific region within all three strains ( Figure 3). This would corroborate the excitation energy transfer to PSI and II in strain HICR111A and could explain the observed ROS production under yellow and green light. However this would also refute previous reports on the absence of phycobiliproteins in this strain ^5^. Given that phycobiliproteins were not purposely extracted, and further analyzed, in the present study, we can at this point only speculate about their presence and relative expression under different growth conditions.

There is a long history of associating pigment compositions within phototrophs with the spectral composition of ambient light and exposure history: for recent work see ^38^ and ^23^, respectively. These two factors are likely to determine the sensitivity of phototrophs to irradiance and their capability to cope with ROS levels generated upon irradiation. Interestingly, we found that strain CRS generated very little ROS upon exposure to VIS when previously acclimated to NIR, while strain MBIC11017 and HICR111A appear to be more sensitive to VIS when previously adapted to NIR ( Figure 2A). HPLC analysis revealed a higher ratio of zeaxanthin/α-carotene in NIR-acclimated CRS cells than in the other two strains ( Table 1), albeit with a relatively large standard error. In *A. marina* MBIC11017 α-carotene was found to be an integral part of both photosystem reaction center cores ^39, 40^ and zeaxanthin is predominantly found in the periphery of light-harvesting complexes ^36^.

Based on the higher zeaxanthin/α-carotene ratios in NIR-adapted cells of all three strains we hypothesize that there are slightly more antenna complexes (zeaxanthin and Chl *d*) in NIR-adapted cells than in those pre-adapted to VIS, thus providing more potential for ROS production/quenching. Given that the antenna complexes are predominantly composed of Chl *d* and thus absorb in the NIR part of the light spectrum, this chromatic photoacclimation is expected and further corroborated by higher Chl *d*/zeaxanthin ratios in NIR-adapted strains in this study. We hypothesize that the higher contribution of zeaxanthin in the NIR-adapted strain CRS could aid in effectively capturing NIR but potentially also in quenching ROS produced during illumination with either VIS or NIR. However, we acknowledge that this hypothesis is based on HPLC analysis of pigment levels with a relatively high variability and one individual ROS measurement.

Besides their light harvesting capability carotenoids (including zeaxanthin) are also known for their antioxidative abilities ^15, 35^. It is known that zeaxanthin mostly operates outside of the reaction centers (RC) and is predominantly found within the peripheral light-harvesting complexes ^35^. Here, zeaxanthin was shown to play a crucial role in non-photochemical quenching and energy dissipation from sensitized chlorophyll molecules or singlet oxygen ^35^. Singlet-oxygen formation is often inevitable and is believed to necessitate the rapid turnover of the photosystem II-D1 polypeptide ^41^. It was found that the PSII-D2 protein acquires a certain photoprotection against singlet-oxygen by close association with β-carotene molecules in the RC of PSII ^42, 43^ and hence has a lower turnover than the D1 protein. We thus hypothesize that the presence of α-carotene in the reaction centers of strain MBIC11017 ^40, 44^ could provide photoprotection in certain strains of *A. marina*. Whether carotenoids are directly involved in ROS quenching remains unknown and thus we can only speculate about the exact levels of ROS being produced at PSI/PSII and within light-harvesting complexes of *A. marina*. Nonetheless, our measurements indicate lower levels of ROS during exposure to NIR when compared to VIS; whether this is related to the replacement of Chl *a* with Chl *d* in *A. marina* remains to be investigated.

Lastly, it is possible that strain specific differences in ROS production are associated with dissimilarities in growth forms: Strain HICR111A forms cell aggregates ^5^, and so does strain CRS ( Figure 2B), whereas strain MBIC11017 usually grows as dispersed cells ^5^ but can be immobilized into biofilms ^45^. The formation of aggregates in strain HICR111A and CRS might provide photoprotection through self-shading, a behavior reportedly less pronounced in strain MBIC11017 ^5^. Both HICR111A and CRS originate from shallow reef flats, a high irradiance habitat. In contrast, strain MBIC11017 was isolated from a didemnid ascidian ^2^, a light environment depleted of VIS but with sufficient NIR ^10– 12^.

Based on these first, preliminary, measurements, we suggest that through utilization of NIR, *Acaryochloris* can avoid a considerable amount of light stress, while harvesting a portion of the electromagnetic radiation spectrum not used by other oxyphototrophs. Additionally, aggregation of certain strains could protect against excess amounts of ROS generated during high irradiance exposure. Overall, this could add further arguments as to why *Acaryochloris* is a successful and apparently globally widespread oxyphototroph ^11^.

## Materials and methods

### Isolation of the novel *Acaryochloris* strain CRS

Dead coral branches with patches of faint yellow-greenish pigmentation were collected during low tide from coral patches on the inner reef flat off Heron Island, Queensland, Australia (see more details on the sampling site in ^10^. The samples were transported back to the lab in a container with seawater and immediately placed into outdoor aquaria that were continuously flushed with aerated ambient seawater pumped in from the reef flat. Bacterial cells found on the dead coral branch were removed using a sterile scalpel and immediately placed into KESM media and kept under dim visible light for three days. After transportation to Sydney the cells were kept in KESM media under NIR LEDs (centered at 720 nm, Cat. No. L720-04AU, Epitex Inc., Japan). NIR irradiance was set to ~5 µmol photons m ^-2^ s ^-1^ using a SKP200 light meter equipped with a SKP216ER irradiance sensor with a 550 to 750 nm light sensitivity range (Skye Instruments, United Kingdom). After three weeks, the growing cells were diluted into aliquots of fresh KESM medium. After additional incubation, the pigmentation of the cells was inspected by measuring their absorption characteristics using a spectrophotometer (UV-2550, Shimadzu, Japan). The cells were hereafter maintained in KESM media under NIR.

### *Acaryochloris* growth conditions

*Acaryochloris* strains MBIC110771, HICR111A and the newly isolated *Acaryochloris* strain CRS were grown in 200 ml cell culture flasks in marine KESM media (salinity of 30) in a shaking incubator at 28°C as previously reported ^1^. All cultures were shaken at 100 rpm under a 12/12 h light-dark shift. Near infrared radiation (NIR) was provided by narrow band LEDs (L720-04AU, 700–740 nm, centered at 720 nm, Epitex Inc., Japan) at an irradiance of 20–40 µmol photons m ^-2^ s ^-1^. Another set of cultures was grown under the same irradiance but using visible light delivered by a halogen lamp equipped with a heat filter (HQ Power, Brinck Elektronik, Denmark). Absolute irradiance measurements of NIR and visible light were done with a calibrated spectroradiometer (Jaz ULM-200, Ocean Optics, Dunedin, FL, USA).

### DNA extraction and PCR amplification

Six ml of dense cell culture was spun down and then extracted using the FastDNA for Soil kit (MP Biomedicals, France) using the manufacturers standard protocol. The resulting DNA was quantified using the Qubit system (Invitrogen, Life Technologies Europe, USA) and diluted 1:10 using molecular grade water. The *16S rRNA* gene was amplified using the primers 16SCYfw (5´-GGCTCAGGATGAACGCTGGCGG-3´) and 16SCYrv (5´-ACCTTGTTACGACTTCACCCCAGTC-3´) using the PCR Master (Roche, Switzerland) with 30 amplification cycles. The resulting PCR product was purified on an agarose gel and the band excised using a sterile scalpel. DNA was extracted from the excised gel using the QiaexII gel extraction kit (Qiagen Nordic, Sweden) and then cloned into the pCR4-TOPO cloning vector (Invitrogen, Life Technologies Europe, USA) and transformed into One-Shot TOP-10 chemically competent cells (Invitrogen, Life Technologies Europe, USA). Clones were subsequently grown in LB-medium, plasmids were extracted using the Qiaprep kit (Qiagen Nordic, Sweden), and checked for correct sized inserts using gel electrophoresis. Three clones were sent off for subsequent sequencing by a commercial provider (Macrogen, Seoul, Korea).

### Phylogenetic analysis

Cyanobacterial *16S rRNA* gene sequences were retrieved from the SILVA database ( http://www.arb-silva.de/) and aligned together with sequences retrieved from clones using MUSCLE as implemented in the Molecular Genetic Analysis (MEGA) software package version 5.0. Neighbor-joining (NJ) was used to infer phylogenetic relationships among sequences; support values with Jukes-Cantor distances and 10000 bootstrap replicates are displayed next to branches displayed in the phylogenetic tree ( Figure 1).

### Chlorophyll extraction and spectrophometry

Two ml of each culture were pelleted by centrifugation at 8000 x g. The supernatant was removed, while the resulting pellet was re-suspended in 96% ethanol and incubated at 4°C for 60 min in darkness. During the ethanol extraction, the samples were vortexed at maximal speed every 15 minutes. After one hour, the cells were pelleted by centrifugation at 8000 x g and the supernatant was used to determine Chl *d* concentrations via spectrophotometry (UV-2101PC, Shimadzu, Japan) according to Ritchie ^46^. The same spectrophotometer was used to measure the *in vivo* absorbance spectra of the different cultures. *Acaryochloris* strains HICR111A and CRS proved very difficult to keep in suspension and were therefore sonicated (Misonix sonicator 4000, Qsonica LLC., Newtown, CT, USA) for one minute at maximum speed prior to spectrophotometric measurements. To prevent bleaching of the photopigments, all handling was done as quickly as possible and under low-light conditions.

### HPLC-based pigment analysis

For HPLC analysis, 2 ml of *Acaryochloris* cultures were spun down at maximum speed (~13,000 rpm) in a bench centrifuge, the supernatant was removed and the remaining pellet resuspended in cold acetone-methanol (7:2 by vol) and the cells sonicated for 20s using a Misonix sonicator 4000 (Qsonica LLC., Newtown, CT, USA) according to ^47^. The cells were incubated for 2 min on ice in complete darkness, centrifuged again and the extract filtered through a Minisart 0.2-µm pore-size filter (Sartorius, Germany). Ammonium acetate (15 µl; 1.0 M) was added to the extracts (150 µl) to further improve pigment resolution before subsequent injection. Pigment separation was performed on an Agilent 1260 infinity HPLC machine (Agilent Technologies, Santa Clara, CA, USA) and a Nova-pak C18 column (dimensions: 3.9 x 300 mm). A 1260 Infinity Multiple Wavelength Detector (Agilent Technologies, Santa Clara, CA, USA) was used for the detection of compound specific absorption wavelengths. *Acaryochloris* culture extracts were run with solvent A (methanol:acetonitrile:water, 42:33:25 by vol) and solvent B (methanol:acetonitrile:ethyl acetate, 39:31:30 by vol) in a gradient comprised of 40% solvent B at time of injection, a linear increase to 100% B at 60 min and back to 40% B in 3 minutes. Flow rate was kept constant at 1 ml min ^-1^ and the column at a temperature of 30°C. Photopigments were identified manually from HPLC chromatograms and ratios calculated based on the derived peak areas. Average values and standard error from the mean from two independent growth experiments were calculated and are displayed in Table 1.

### ROS measurements via real-time ethylene detection

Real-time ethylene production was measured using a laser-based photo-acoustic ethylene detector (ETD-300, Sensor Sense, the Netherlands) combined with an in-line gas-flow through system (Valve Controller VC 6, Sensor Sense, the Netherlands). The system was described in reference ^26^. Custom made gas-tight incubation chambers were connected via the valve controller to the ETD, which could sequentially sample ethylene fluxes from the different incubators. The incubator was made of anodized aluminum (51ST quality) and contained a cooling/heating channel to control temperature and a glass window to supply light to the samples (see details in ^48^). The incubator could hold 2 ml aliquots of *Acaryochloris* culture. Culture samples were augmented with α-keto-γ-(methylthio)butyric acid sodium salt (KMBA) (<97% purity, K6000, Sigma-Aldrich) made to a final concentration of 2.8 mM in KESM media; earlier experiments on cyanobacterial cultures demonstrated saturating levels of KMBA when supplied at this concentration (Staal *et al.*, unpublished). After addition of KMBA, the cultures were mixed using a pipette to obtain a uniform distribution of the chemical. All measured ethylene concentrations were normalized to the Chl *d* concentration in the samples.

To ensure steady state ethylene fluxes at the moment a sample was connected to the ETD, we supplied a continuous flow of moisturized air (2 l h ^-1^) over every individual incubator during the experiments. The air was moisturized by flushing it through gas tight vials filled with de-ionized water; this was necessary to prevent evaporation of media in the incubator. The system was continuously controlled for gas leaks, by automated comparison of the incoming and outgoing gas flow. The outlet of the incubator was connected to a CO _2_ trap (KOH pellets) and water scrubber (CaCl _2_) placed before the ethylene detector. The valve controller allowed each measuring chamber to be alternately connected for 20 minutes to the ethylene detector. Steady state ETD readings from the cultures were obtained within ~4 minutes after connection to the ETD. The ETD-300 has a sample frequency of ~12 samples min ^-1^ and the concentrations of the last two minutes per treatment were averaged. Typical standard deviations were 0.15 ppbv for ethylene measurements under steady state conditions. The averaged concentrations were normalized to the amount of Chl *d* present in the cultures to correct for differences in biomass between samples.

### Light experiments

Light dependent ROS production was measured using both visible light (400–700 nm) and near-infrared radiation (NIR). For visible wavelengths, we used an incubator setup (Mini-Incubator, Sensor Sense, Nijmegen, The Netherlands), fitted with an array of 11 1W cool white LEDs (Luxeon Star, 1W, Lumileds, USA) connected to a PC-driven controller. Irradiance levels were set between 340–480 µmol photons m ^-2^ s ^-1^ for visible light. Different irradiance levels were adjusted by varying the electrical current of the LED array via a special software routine (Sensor Sense, Nijmegen, The Netherlands) and measuring the downwelling irradiance with a calibrated light meter (LI250, LiCOR Biosciences, Lincoln, USA).

For NIR exposure, the actinic light was provided by four collimated NIR LEDs (M3L1-720–30, 700–740 nm, centered at 720 nm, Roithner Lasertechnik, Vienna, Austria) at an intensity of 400 µmol photons m ^-2^ s ^-1^. The absolute NIR irradiance level was measured with an irradiance sensor attached to a calibrated spectroradiometer (Jaz ULM-200, Ocean Optics, USA). Subsequently, the measured irradiance spectrum was integrated over a spectral range of 650–800 nm. All samples were incubated at 28°C using a cooler/heater bath (HD-25, Julabo, Germany).

Action spectra of ROS production were measured using three high-power LEDs (Luxeon star, 1W, Lumileds, USA) mounted onto an aluminum plate for efficient cooling. The following colors and centered wavelengths were used in the action spectra: Blue (470 nm, 25 nm spectral half-width, LXHL-MB1D), Cyan (495 nm, 30 nm spectral half-width, LXHL-ME1D), Green (535 nm, 35 nm spectral half-width, LXHL-MM1D), Amber (595 nm, 14 nm spectral half-width, LXHL-ML1D), Red (645 nm, 20 nm spectral half-width, LXHL-MD1D). The LED’s were powered by LED power supplies (LED31, Velleman, Belgium). For each LED color, the incident irradiance was adjusted to 300 µmol photons m ^-2^ s ^-1^ by adjusting the distance from the LEDs to the incubation chamber window. Photon irradiances were measured with a calibrated light meter (LI250, LiCOR Biosciences, Lincoln USA) before each measurement.

To the best of our knowledge we are the first to provide data on ROS levels associated with NIR-driven oxygenic photosynthesis. Unfortunately, due to measuring time constrains at the trace gas facility we were unable to provide replicated measurements of ROS levels under various light regimes. This emphasizes the need to perform additional measurements of NIR induced ROS production in *A. marina* and other far-red utilizing oxyphototrophs.
