# Supplementary material for: Reactive oxygen production induced by near-infrared radiation in three strains of the Chl d-containing cyanobacterium Acaryochloris marina
Source: F1000Res. 2013 Mar 13;2:44. Originally published 2013 Feb 13. [Version 2] doi: 10.12688/f1000research.2-44.v2 (PMC3894803; doi:10.12688/f1000research.2-44.v2)
Supplement: Peer review history

# Peer review: Referee response for version 2

**DOI:** 10.5256/f1000research.1267.r3978

**Published:** 2014-03-05

**Authors:** Helen Yap

**Affiliations:** 1 The Marine Science Institute, University of the Philippines, Quezon City, Philippines

I have read this submission. I believe that I have an appropriate level of expertise to confirm that it is of an acceptable scientific standard.

# Peer review: Referee response for version 2

**DOI:** 10.5256/f1000research.1267.r3184

**Published:** 2014-01-17

**Authors:** Susanna López Legentil

**Affiliations:** 1 Department of Animal Biology, Universitat de Barcelona, Barcelona, Spain

I have read this submission. I believe that I have an appropriate level of expertise to confirm that it is of an acceptable scientific standard.

# Peer review: Referee response for version 1

**DOI:** 10.5256/f1000research.868.r795

**Published:** 2013-02-22

**Authors:** Alison Telfer

**Affiliations:** 1 Wolfson Laboratories, Imperial College London, London, UK

This paper shows experiments on several cyanobacterial strains of the *Acaryochloris* genus, in which chlorophyll *a* is replaced by Chl *d* which absorbs in the near infra red (NIR) - 25-30 nm to the red of Chl *a*. It shows that under stress levels of NIR excitation (and also adaptation to growth under NIR) less reactive oxygen species (ROS) are formed. The authors conclude that this is a strategy of this genus (more species of which are being discovered widely around the planet) to protect against photodamage during high irradiance exposure - a more and more likely occurrence these days.

The authors stress the point (page 7, left, para 3) that this is a preliminary study in which fully replicated measurements of ROS were not possible. I understand this constraint and accept that reporting measurements of ROS, using the novel technique of real time ethylene detection, from *Acaryochloris* is very interesting but I feel the paper goes too far in its claims. It also is unclear about the different types of ROS detected and the mechanism by which protection is provided by carotenoids.3, right para 2.

- The ROS detection method (real time ethylene detection RTED) described in ref 28 (Regoli and Winston) appears to only directly detect very strong oxidants hydroxyl and peroxyl radicals and peroxynitrite. It does not directly detect superoxide or singlet oxygen (1O2). This should be made clearer in the text and there should be discussion about production of hydroxyl radicals. 
- Relating to point 1.: The correlation between light-induced increase in relative amounts of the zeaxanthin containing antenna complexes and the ROS levels detected by the RTED system should be explained in more detail. Carotenoids in photosynthetic complexes mainly operate by quenching chlorophyll triplet states before they can form 1O2 or they directly quench any 1O2 that is formed. They do also quench oxygen radicals but these are more likely to be produced in the aqueous phase (e.g. from reduced ferredoxin in PSI) where they are usually quenched by antioxidant enzymes such as ascorbate peroxidase etc. Though it is possible alpha-Car in the PSII reaction center could quench radicals produced from reduced quinone. When the ROS levels, detected by RTED, increase it is likely that other ROS (e.g. superoxide) and perhaps 1O2 also increase and so carotenoids would be helpful. However, the text makes it sound as if in CRS the zeaxanthin level increases relative to chlorophyll when it is simply more antenna (Zea plus Chl *d*) being produced and so there is more potential for 1O2 production.
- Essentially I feel the text, though cautious, claims too much. The errors on the pigment levels especially for CRS (they are huge) suggest the very different values for NIR and VIS adapted cells could be a fluke. Also the single point for CRS in Fig. 2A NIR adapted under VIS exposure could be a fluke.

**Minor Points**

p. 4 end para beginning ‘In *Acaryochloris*....:   Change last sentence round so it says: zeaxanthin will bring about rapid quenching of excited chlorophyll states and if necessary can also quench singlet oxygen - or something similar to this.

p.6 end para 3:   Rephrase: ...and zeaxanthin is restricted to the peripheral light-harvesting complexes (PCB proteins).

p.6 Second sentence from bottom: I do not like the sentence emphasising that zeaxanthin quenches singlet oxygen. The a-carotene in the reaction centre is at least as likely to be quenching singlet oxygen as it is well known it cannot directly quench the radical pair triplet state as it is bound to far away from the highly oxidising primary electron donor. The reference to energy dissipation by zeaxanthin is irrelevant here. 

Fig. 2C   For clarity use different symbols (but same colour) for NIR adapted and VIS adapted points.

I have read this submission. I believe that I have an appropriate level of expertise to confirm that it is of an acceptable scientific standard, however I have significant reservations, as outlined above.

# Peer review: Referee response for version 1

**DOI:** 10.5256/f1000research.868.r778

**Published:** 2013-02-18

**Authors:** Susanna López Legentil

**Affiliations:** 1 Department of Animal Biology, Universitat de Barcelona, Barcelona, Spain

**Overall evaluation**: In this manuscript, Behrendt and coworkers present some novel results about the generation of reactive oxygen species (ROS) by a cyanobacterium ( *Acaryochloris marina*) that relies on chlorophyll *d* for photosynthesis. Although I am not an expert on the biochemical pathways involved in photosynthesis, the methodology chosen appears appropriate to address the question at hand and the experiments appear to have been well conducted. Additional replicates and tests are still necessary to confirm the pattern found, but I believe the subject matter addressed in this manuscript and the results obtained are of enough interest to warrant immediate publication.

**Limitation of the study**: As the authors point out, additional measurements of ROS levels under various light regimes are necessary. Data should also be subjected to appropriate statistical analyses to derive stronger conclusions. In addition, it will be interesting to test another strain that does not aggregate to reinforce some of the conclusions presented in the article. 

**Introduction**: In the first paragraph you mentioned that 2 strains of *A. marina* have been well described, but then proceed to describe only one (MBIC11017). Please, add some information about the second well-described strain.

I have read this submission. I believe that I have an appropriate level of expertise to confirm that it is of an acceptable scientific standard.

# Peer review: Referee response for version 1

**DOI:** 10.5256/f1000research.868.r776

**Published:** 2013-02-18

**Authors:** Helen Yap

**Affiliations:** 1 The Marine Science Institute, University of the Philippines, Quezon City, Philippines

**Overall evaluation**: This is a well-written paper describing potentially ground-breaking research which has universal application, considering the ubiquitous occurrence of the cyanobacteria studied, and the crucial role they play in primary production of shallow-water habitats worldwide.

**Limitation of the study**: The authors acknowledge that the experiment suffers from lack of replication of measurements of ROS levels under the different light regimes. They also do not include statistical analyses that would have confirmed significant differences among the different strains, and between treatments (NIR- and VIS-adapted).

Nevertheless, the results are interesting and merit further investigation. In general, the experiment appears to have been conducted following appropriate scientific standards and protocols, and using valid methods. 

**Results and Discussion**:3rd from last paragraph: “concretionary” is not a common term to define coral reef substrata

I have read this submission. I believe that I have an appropriate level of expertise to confirm that it is of an acceptable scientific standard.
